# Myeloid-Derived Suppressor Cells Specifically Suppress IFN-γ Production and Antitumor Cytotoxic Activity of Vδ2 T Cells

**DOI:** 10.3389/fimmu.2018.01271

**Published:** 2018-06-06

**Authors:** Alessandra Sacchi, Nicola Tumino, Andrea Sabatini, Eleonora Cimini, Rita Casetti, Veronica Bordoni, Germana Grassi, Chiara Agrati

**Affiliations:** Laboratory of Cellular Immunology and Pharmacology, Department of Epidemiology, Pre-Clinical Research and Advanced Diagnostic, National Institute for Infectious Diseases “Lazzaro Spallanzani” IRCCS, Rome, Italy

**Keywords:** γδ T cells, myeloid-derived suppressor cells, antitumoral activity, immunotherapy, IFN-γ

## Abstract

γδ T cells represent less than 5% of circulating T cells; they exert a potent cytotoxic function against tumor or infected cells and secrete cytokines like conventional αβ T cells. As αβ T cells γδ T cells reside in the typical T cell compartments (the lymph nodes and spleen), but are more widely distributed in tissues throughout the body. For these reasons, some investigators are exploring the possibility of immunotherapies aimed to expand and activate Vδ2 T cells, or using them as Chimeric Antigen Receptor carriers. However, the role of immunosuppressive microenvironment on Vδ2 T cells during infections and cancers has not been completely elucidated. In particular, the effects of myeloid-derived suppressor cells (MDSC), largely expanded in such pathologies, were not explored. In the present work, we demonstrated that MDSC may inhibit IFN-γ production and degranulation of phosphoantigen-activated Vδ2 T cells. Moreover, the Vδ2 T cells cytotoxic activity against the Burkitt lymphoma cell line Daudi and Jurkat cell line were impaired by MDSC. The Arginase I seems to be involved in the impairment of Vδ2 T cell function induced by both tumor cells and MDSC. These data open a key issue in the context of Vδ2-targeted immunoteraphy, suggesting the need of combined strategies aimed to boost Vδ2 T cells circumventing tumor- and MDSC-induced Vδ2 T cells suppression.

## Introduction

γδ T cells represent between 2 and 10% of total circulating CD3+ T lymphocytes (1). Among peripheral CD3+ γδ T cells, those expressing a TCR formed by the Vγ9 and Vδ2 variable regions (hereafter referred to as Vδ2 T cells) constitute up to 90% of γδ T cells (2). These Vδ2 T cells specifically recognize non-peptidic phosphorylated metabolites of isoprenoid biosynthesis, present in most pathogenic bacteria (3), or isopentenyl pyrophosphate, produced also by the human mevalonate biosynthesis pathway (4) that get accumulated in virus-infected and cancer cells due to alterations in the mevalonate pathway. Vδ2 T cells are characterized by high plasticity in their functions: they have the ability to produce pro-inflammatory cytokines, such as IFN-γ, TNF-α (5), and IL-17 (6) and also act as professional antigen-presenting cells (7). Further, Vδ2 T cells act as a bridge between the innate and adaptive immune response, inducing dendritic cell (DC) maturation and shaping the Th1 and cytotoxic CD8 T cells response (8). Stimulated Vδ2 T cells also display strong cytolytic activity toward allogeneic tumors of various tissue origins. In particular, Vδ2 T cells are able to infiltrate murine and human tumors, to recognize tumor antigens, and to secrete cytotoxic molecules like granzyme and perforin (9, 10). The wide antitumor activity as well as their ability to shape both innate and adaptive immune response in the tumor microenvironment make these cells as good target in immunomodulating strategies in cancer settings. Several clinical trials of Vδ2 T cell-based immunotherapy performed for both hematological malignancies and solid tumors show promise for cellular therapy to control cancer (11); however, the effectiveness of Vδ2 T cell-based therapy is limited, probably due to suppressive function of the tumor microenvironment.

Myeloid-derived suppressor cells (MDSC) are potently immunosuppressive myeloid cells that accumulate in patients with advanced cancers (12), and in various disease settings, including chronic inflammation, viral infections (such as HIV infection), and autoimmune diseases (13–16). At present, it is well known that there are at least two main subsets of MDSC: the monocytic (Mo-MDSC) and the granulocytic (PMN-MDSC) subsets. In particular, in humans PMN-MDSC are identify as CD14− CD11b+ HLA-DRlow/− CD15+ (or CD66+), while Mo-MDSC as CD11b+ CD14+ HLA-DRlow/− CD15− (17), both subsets express CD33 myeloid marker. MDSC suppress T cell activation and cytotoxicity, induce the differentiation and expansion of Tregs, and inhibit NK cell activation by using different mechanisms [reviewed in Ref. (18)]. However, little is known about the suppressive activity of MDSC on Vδ2 T cells. It has been demonstrated that during HIV infection PMN-MDSC are expanded and their frequency is inversely correlated with the capacity of Vδ2 T cells to produce IFN-γ. However, differently from αβ T cells, *in vitro* PMN-MDSC depletion did not completely restore IFN-γ production by Vδ2 T cells from HIV patients (13), suggesting that during HIV infection PMN-MDSC are not the unique player in dampening Vδ2 T cell response. Thus the exact role of MDSC in regulating Vδ2 T cells functions remains to be elucidated. Aim of the present work was to shed light on the effects of the suppressive capacity of MDSC on Vδ2 T cells capabilities.

## Materials and Methods

### Peripheral Blood Mononuclear Cells (PBMC) Separation

PBMC were obtained from buffy coats kindly provided from S. Camillo Hospital. According to NIH definition (https://humansubjects.nih.gov), this study does not require Ethical Committee approval. PBMC were isolated from peripheral blood by density gradient centrifugation (Lympholyte-H; Cederlane). After separation, PBMC were resuspended in RPMI 1640 (EuroClone) supplemented with 10% heat-inactivated fetal bovine serum (EuroClone), 2 mmol/L l-glutamine, 10 mmol/L HEPES buffer (*N*-2-hydroxyethylpiperazine-*N*-2-ethane sulfonic acid) and with 2 mmol/L penicillin and 50 µg/mL streptomycin (EuroClone), hereafter indicated as R10.

### Cell Subsets Purification

**Figure 1:**
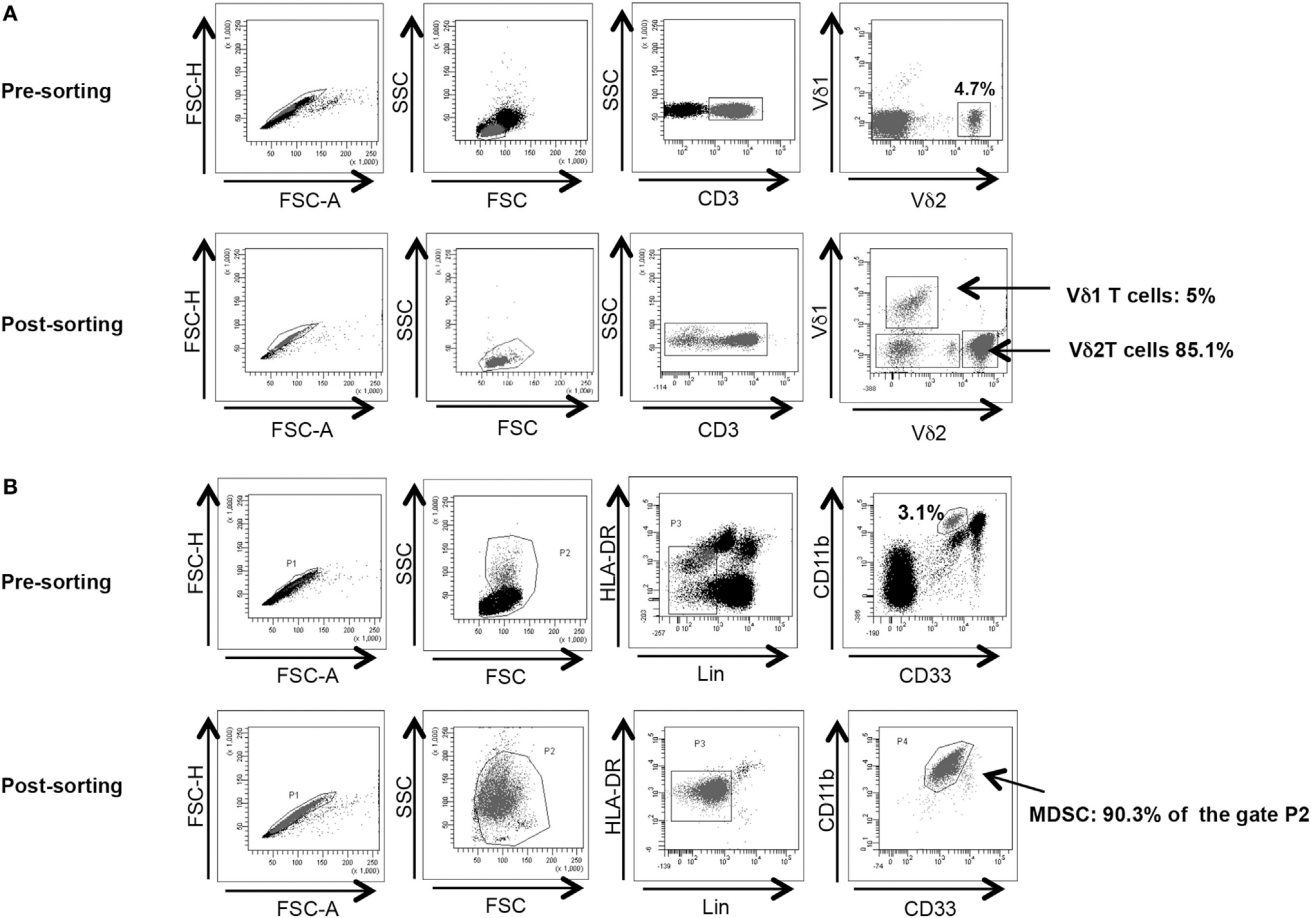
γδ T cell and PMN-myeloid-derived suppressor cells (MDSC) purity after magnetic sorting. **(A)** Representative purity of γδ T cells before and after sorting. Gating strategy is shown. **(B)** Representative purity of PMN-MDSC before and after sorting. Gating strategy is shown.

Vδ2 T cells and PMN-MDSC frequency were analyzed before cell sorting. To obtain a better purification, when Vδ2 T cell and PMN-MDSC percentages were higher than 2 and 3%, respectively, we proceeded to cell sorting as follow: γδ T cells were purified from PBMC using a magnetic negative selection (TCRγδ + T cells isolation kit, Miltenyi Biotec) according to manufacturer’s instruction. PMN-MDSC isolation from PBMC using CD15 microbeads (Miltenyi Biotec) according to manufacturer’s protocol. The purity of sorted γδ T cells and PMN-MDSC was >85 and 90%, respectively, as verified by flow cytometry (Figures 1A,B respectively). Purified cells were rested for 18 h in R10.

### Cell Stimulation

Cytokine production and degranulation by γδ T cells was tested by stimulating cells with IPH11 (3 µM, Innate Pharma) in the presence of PMN-MDSC. Brefeldin A was added after 1 h of stimulation. After 18 h, IFN-γ and CD107a were evaluated by flow cytometry. Titration of PMN-MDSC/γδ T cells ratio was performed (2:1, 1:1, 1:2, 1:5) evaluating CD107a expression.

Cytotoxic ability of Vδ2 T cells was analyzed using Daudi and Jurkat cell lines as targets and measuring annexin V binding to apoptotic cells. Briefly, titration of Daudi cells and Jurkat cells/γδ T cells ratios were performed (2:1, 1:1, 1:2, 1:5, 1:10 and 1:1, 1:2, 1:5, 1:10, 1:20, respectively) testing the binding of annexin V (Annexin V-FITC Apoptosis Detection Kit, eBiosciences) to apoptotic Daudi or Jurkat cells by flow cytometry after 18 h of culture. The percentage of killing was calculated as follow: [100% − (% of living Daudi (or Jurkat) cells in the presence of γδ T cells/% of living Daudi (or Jurkat) cells without γδ T cells) × 100]. Where indicated, specific inhibitors of Arginase I (*N*-Hydroxy-nor-l-arginine, nor-NOHA, 1 mM, Calbiochem), indoleamine 2,3-dioxygenase (1-Methyl-d-tryptophan, 1-MTD, 1 mM, Sigma-Aldrich) or nitric oxide synthases (*N*-Monomethyl-l-arginene, Monoacetate Salt, l-NMMA, 1 mM, Calbiochem) were used.

In order to evaluate MDSC capacity to inhibit T cell proliferation, PBMC or purified γδ T cells were labeled with CFDA-SE (Vibrant CFDA SE cell tracer kit, Invitrogen) according with manufacturer’s instruction. Labeled PBMC were then cultured with purified PMN-MDSC at 1:1 ratio and stimulated with *Staphylococcus* enterotoxin B (SEB, 200 ng/mL, Sigma-Aldrich). CFDA-SE labeled purified γδ T cells were seeded with PMN-MDSC (1:1 ratio) and activated with IPH 11 (3 µM, Innate Pharma) or with the Burkitt lymphoma cell line Daudi (2:1 ratio effector:target) and IL-2 (100 U/mL, Sigma-Aldrich). Cells were maintained at 37°C in humidified air with 5% CO_2_. After 5 days, lymphocytes proliferation was evaluated by flow cytometry.

### Flow Cytometry

The Vδ2 T cells and PMN-MDSC frequency and phenotype were evaluated utilizing the following monoclonal antibodies: anti-Vδ1 (Life technology), anti-NKG2A, anti-NKG2D (Beckman Coulter), anti-NKG2C (R&D system), anti-Vδ2, anti-CD3, anti-CD15, anti-CD33, anti-HLA-DR, cocktail of antibodies anti-CD3, -CD56, -CD19, anti-CD14, anti-CD11b (BD Biosciences). In brief, the cells were washed twice in PBS, 1% BSA, and 0.1% sodium azide and were stained with the mAbs for 15 min at 4°C. The cells were then washed and fixed with 1% paraformaldehyde and analyzed using a FACS Canto II (Becton Dickinson). For intracellular staining, membrane staining was performed as above described. After fixation cells were incubated with anti-IFNγ (BD Biosciences) for 30 min at room temperature. CD107a detection was accomplished by antibody staining during cell stimulation. After washing cells were analyzed using a FACS Canto II (Becton Dickinson). Apoptosis induction of Daudi cells were accomplished by evaluating Annexin V ligation to Daudi (Annexin V-FITC Apoptosis Detection Kit, eBiosciences) following the manufacturer’s instruction. Then cells were stained with anti-CD19, anti-Vδ2, anti-CD3, anti-CD15.

### Statistical Analysis

Results were evaluated using a paired *t* test. A *p* value < 0.05 was considered statistically significant. GraphPad Prism software (version 4.00 for Windows; GraphPad) was used to perform the analysis and graphs.

## Results

### Vδ2 T Cells Are Partially Inhibited by PMN-MDSC

**Figure 2:**
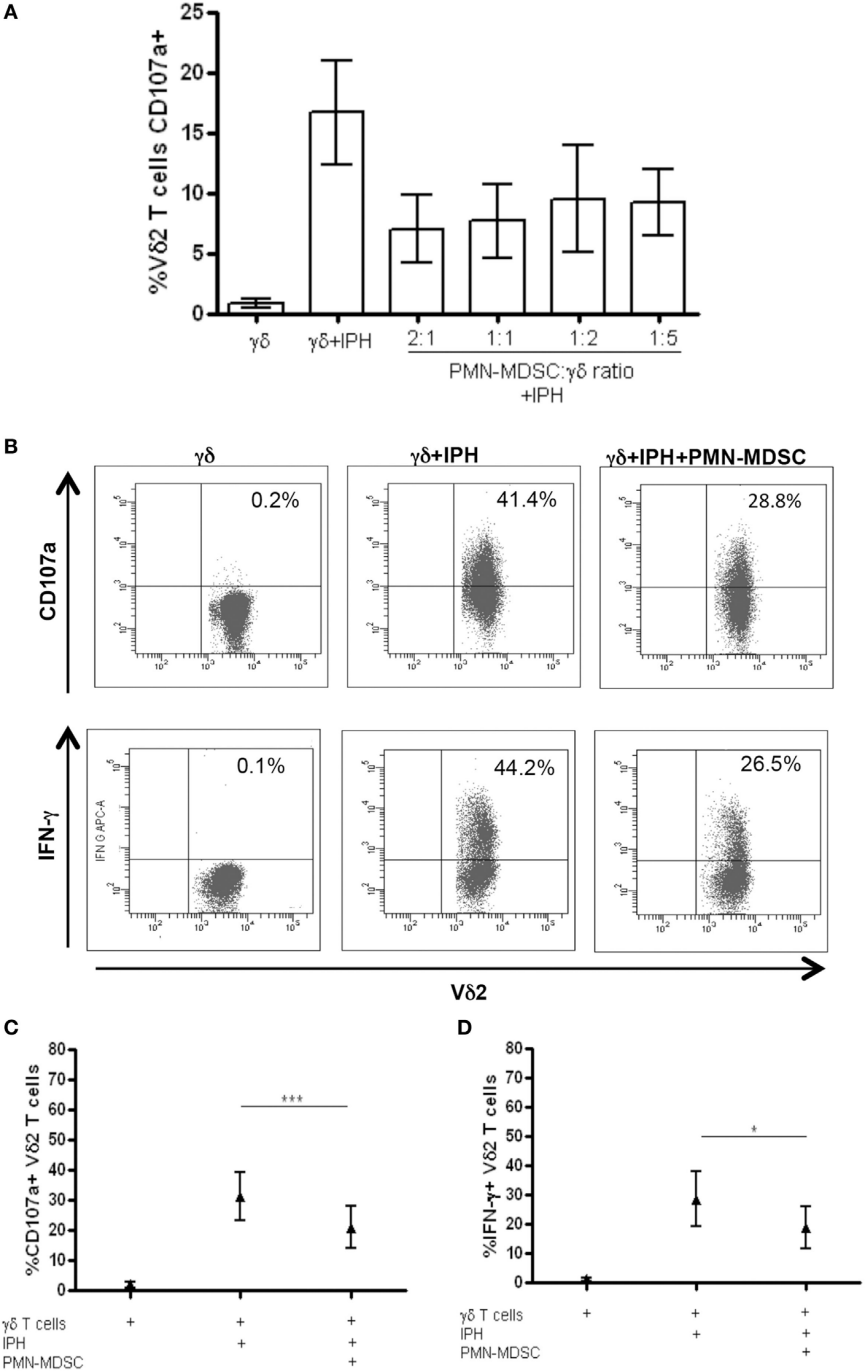
PMN-myeloid-derived suppressor cells (MDSC) inhibit IFN-γ production and CD107a expression by Vδ2 T cells. Purified γδ T cells were stimulated with IPH and IL-2 in the presence of PMN-MDSC and after 18 h IFN-γ and CD107a were evaluated by flowcytometry. **(A)** Titration of PMN-MDSC/γδ T cells ratio tested by measuring CD107a expression on Vδ2+ T cells after IPH stimulation by flowcytometry. Results are shown as mean + SEM of two independent experiments. **(B)** Representative dot plots of IFN-γ production and CD107a expression by Vδ2 T cells. **(C)** Percentage of CD107a + Vδ2 T cells and **(D)** IFN-γ + Vδ2 T cells and in the indicated conditions. Results are shown as Mean + SEM of five independent experiments (*p < 0.05, ***p < 0.0001).

It has been demonstrated that MDSC are able to inhibit T cell activity, but little is known about MDSC/Vδ2 T cell relationship. To address this issue PMN-MDSC and γδ T cells were magnetically purified (purity >90 and >85%, respectively, Figures 1A,B) and were cocultured at different ratios. The ability of MDSC to modulate Vδ2 T cell cytotoxicity and IFN-γ production was evaluated by analyzing the expression of CD107a or IFN-γ on Vδ2 T cells after 18 h. In two preliminary experiments, we optimize the Vδ2/MDSC ratio by looking at CD107a modulation on Vδ2 T cells. As shown in Figure 2A, PMN-MDSC partially inhibit the capacity of Vδ2 T cells to express CD107a in response to IPH stimulation at all ratios (Figure 2A). Therefore, the γδ T cells/PMN-MDSC 1:1 ratio has been used in subsequent five independent experiments, confirming that PMN-MDSC were able to decrease CD107a expression on Vδ2 T cells (Figures 2B,C). We also tested the capability of PMN-MDSC to interfere with IFN-γ production. To this aim, we cultured purified PMN-MDSC and γδ T cells at 1:1 ratio and after 18 h of stimulation with IPH the production of IFN-γ was evaluated by flow cytometry. A decrease of IFN-γ expression was observed in the presence of PMN-MDSC (Figures 2B,D), suggesting that PMN-MDSC may also inhibit cytokine production capacity of Vδ2 T cells.

**Figure 3:**
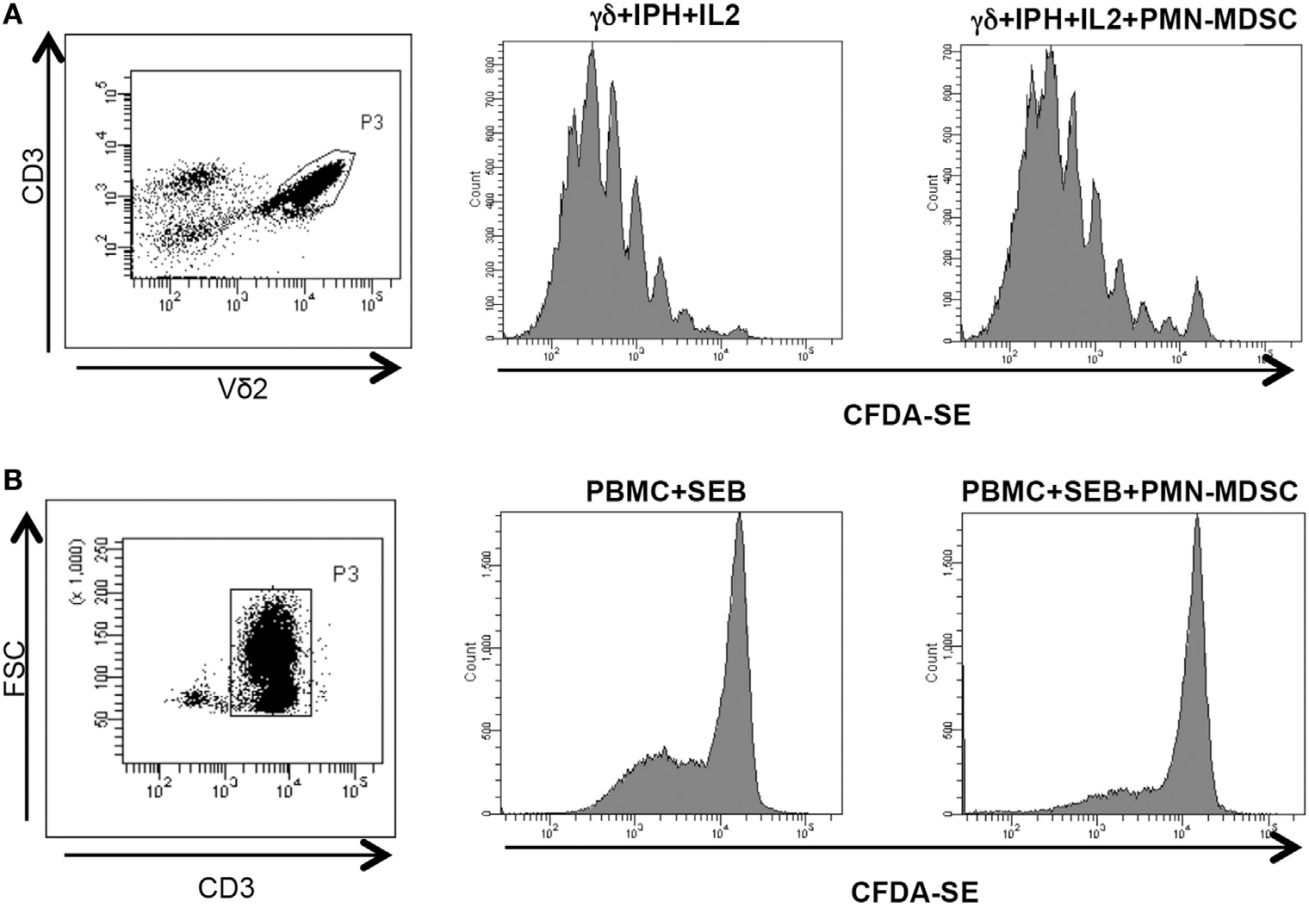
PMN-myeloid-derived suppressor cells (MDSC) does not inhibit Vδ2 T cell proliferation. γδ T cells or peripheral blood mononuclear cell (PBMC) labeled with CFDA-SE were stimulated with IPH in the presence of PMN-MDSC (1:1 ratio). After 5 days, Vδ2+ and CD3+ T cells proliferation was evaluated by flow cytometry. Representative gating strategy and histogram plots of one out of three independent experiments showing Vδ2 T cells **(A)** and CD3+ T cells **(B)** proliferation in the indicated conditions.

We wondered whether PMN-MDSC may impair the proliferation capability of Vδ2 T cells. To this aim, purified CFDA-SE labeled Vδ2 T cells were stimulated with IPH and IL-2 for 5 days and the proliferation rate was evaluated by flow cytometry. We found that PMN-MDSC did not impact the proliferation of Vδ2 T cells (Figure 3A). On the contrary, as previously demonstrated (19) they are able to inhibit the CD3+ T cell proliferation, as SEB-induced proliferation of CD3+ T cells (evaluated using CFDA-SE labeled total PBMC) was decreased by PMN-MDSC (Figure 3B).

### PMN-MDSC Inhibit Vδ2 T Cells Cytotoxicity Against Tumor Cell Lines

**Figure 4:**
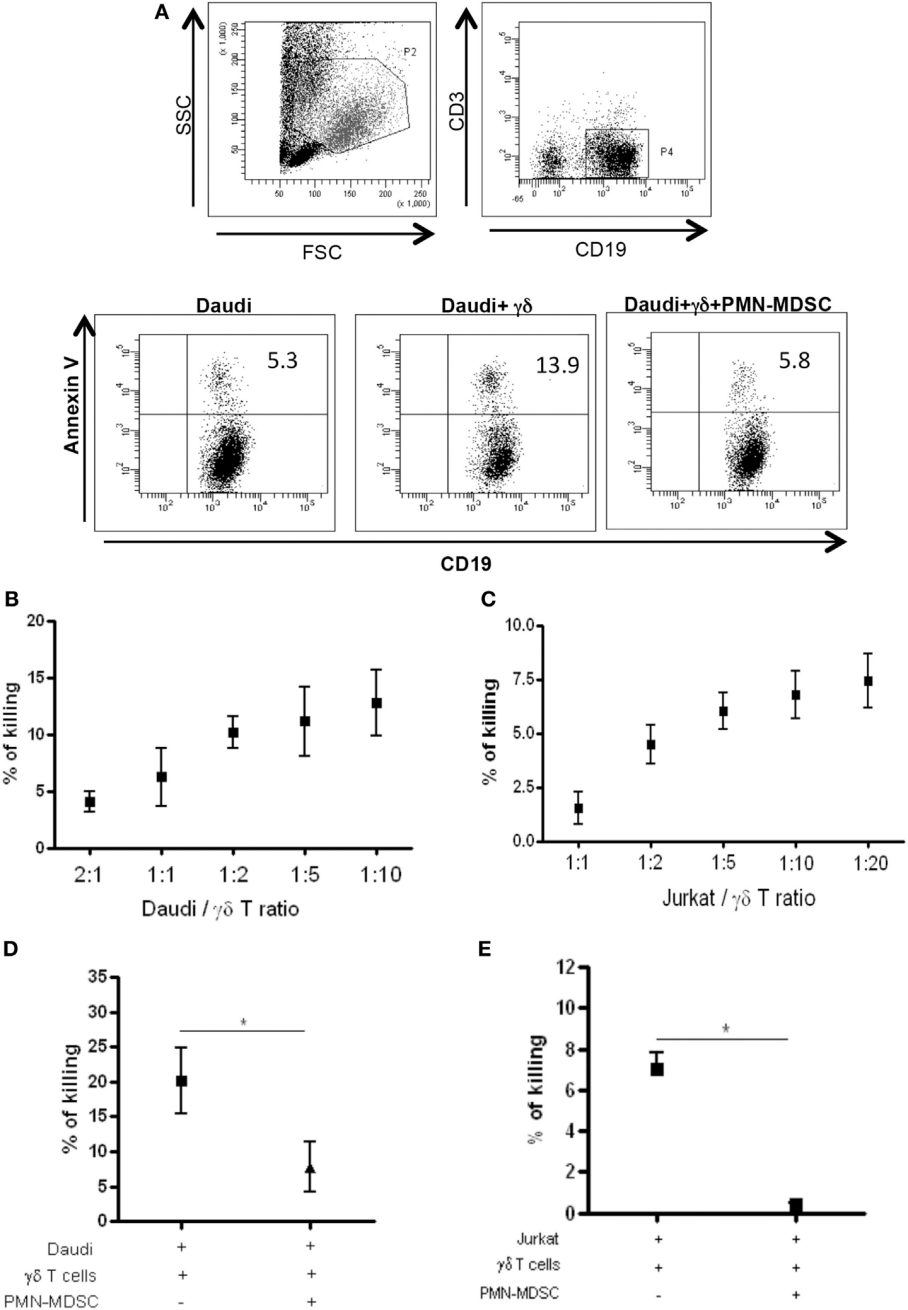
PMN-myeloid-derived suppressor cells (MDSC) inhibit Daudi and Jurkat cells killing by Vδ2 T cells. Daudi and Jurkat cells were cultured with purified γδ T cells and PMN-MDSC, and γδ T cells induced apoptosis were evaluated by measuring annexin V ligation to apoptotic cells. **(A)** Representative dot plots showing gating strategy and Annexin V + Daudi cells in the indicated conditions (1:2:2 ratio). **(B)** and **(C)** Titration of Daudi and Jurkat cells/γδ T cells ratio by measuring the percentage of killing of target cells by flowcytometry. Results are shown as mean + SEM of three independent experiments. **(D)** Percentage of killing of Daudi cells by γδ T cells in the presence of PMN-MDSC (1:2:2 ratio). Results are shown as Mean + SEM of four independent experiments. **(E)** Percentage of killing of Jurkat cells by γδ T cells in the presence of PMN-MDC (1:5:5 ratio). Results are shown as Mean + SEM of three independent experiments (*p < 0.05).

**Figure 5:**
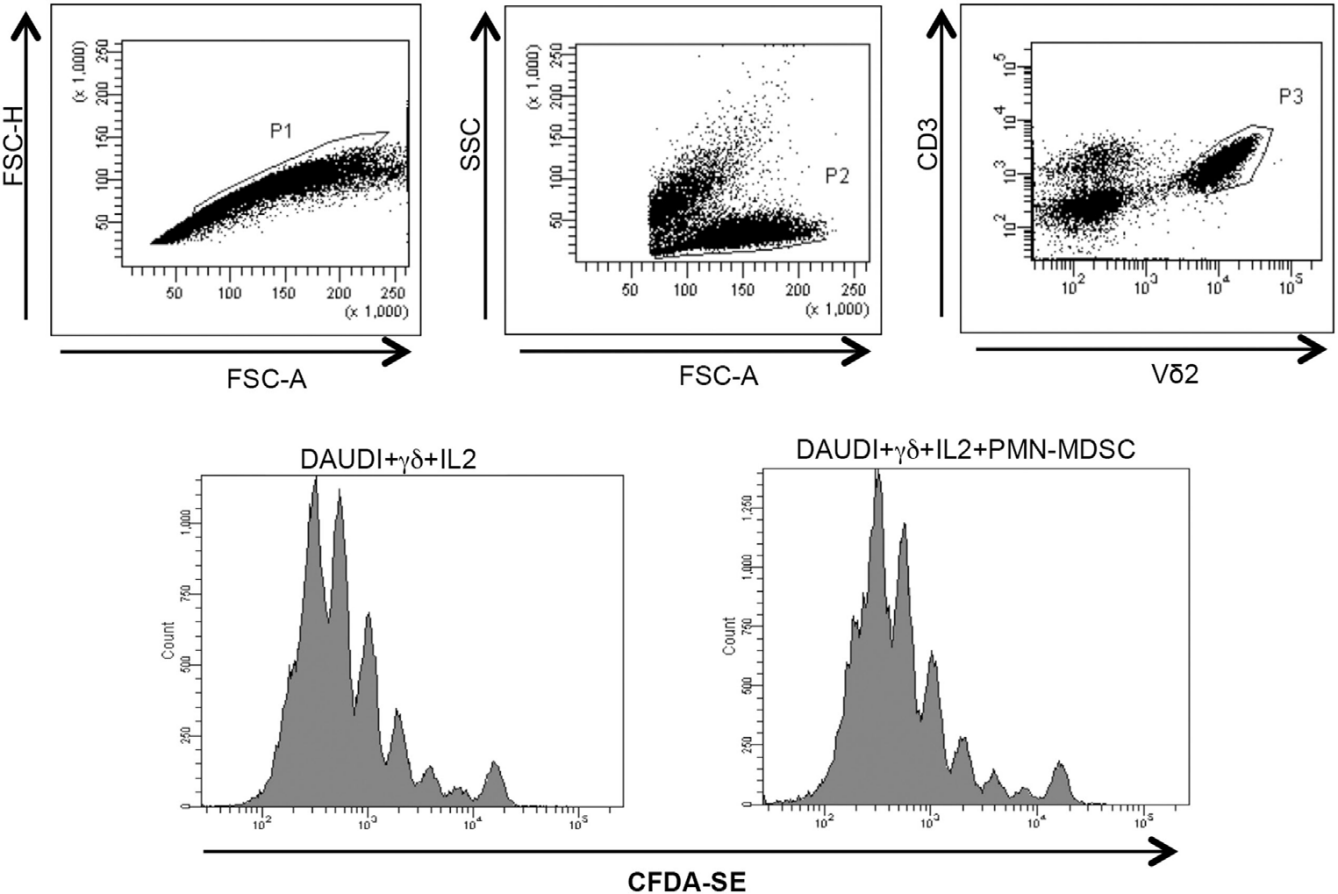
PMN-myeloid-derived suppressor cells (MDSC) does not inhibit Daudi-induced Vδ2 T cell proliferation. Purified γδ T cells labeled with CFDA-SE were stimulated with Daudi cells and IL-2 in the presence of PMN-MDSC. After 5 days, Vδ2+ T cells proliferation was evaluated by flow cytometry. Representative histogram plots of one out of three independent experiments showing Vδ2 T cells proliferation in the indicated conditions.

Vδ2 T cells play a pivotal role in controlling tumor cells exerting a potent cytotoxic function. We wondered if the PMN-MDSC-induced down-modulation of CD107a was correlated to a decrease cytolytic capability towards tumor cells. To answer this question, we first cultured Daudi cells, a Burkitt’s lymphoma known to be a target for Vδ2 T cell cytotoxicity, with purified γδ T cells at different ratios (to select the best ratio) and the cytotoxic activity of Vδ2 T cells was evaluated by analyzing membrane Annexin V ligation on apoptotic Daudi cells by flow cytometry. We found that 1:2 ratio was sufficient to induce Daudi cell apoptosis (Figures 4A,B). Next, Daudi cells were cultured with purified γδ T cells and PMN-MDSC (1:2:2 ratio) for 18 h and apoptotic Daudi cells were analyzed. As expected, we found that Vδ2 T cells are able to kill Daudi cells; however, the presence of PMN-MDSC strongly inhibit their cytotoxic function (Figure 4C). We performed the same experiments with a different tumor cells line (Jurkat), and after assessing the ratio sufficient to induce Jurkat cell apoptosis (1:5, Figure 4C), a decresed capacity of Vδ2 T cells to kill Jurkat were also observed when PMN-MDSC (1:5:5) were added to the culture (Figure 4E). These data indicate that PMN-MDSC inhibition of cytotoxic activity of Vδ2 T cells is not restricted towards Daudi cells. Vδ2 T cell cytotoxicity was deeply controlled by NK receptors expression (20). To evaluate whether MDSC-induced inhibition of Vδ2 T cell cytotoxicity could be mediated by NKRs modulation, the expression of NKG2A, NKG2C, and NKG2D on Vδ2 T cells was evaluated in the absence and in the presence of PMN-MDSC. Results showed that PMN-MDSC did not alter the expression of NKG2A (66.2 ± 8.6 vs 66.3 ± 8.4%), NKG2C (mfi 772 ± 82 vs 748 ± 101), and NKG2D (mfi 1,467 ± 414 vs 1,548 ± 560) on Vδ2 T cells. Finally, Daudi cells were able to induce Vδ2 T cell proliferation in the presence of IL-2, and the proliferation rate of Vδ2 T cells was not affected by PMN-MDSC (Figure 5), confirming that PMN-MDSC do not impair Vδ2 T cells proliferating capacity.

### Specific Inhibitor of ArgI Restore γδ T Cells Cytotoxic Activity Against Daudi Cells

**Figure 6:**
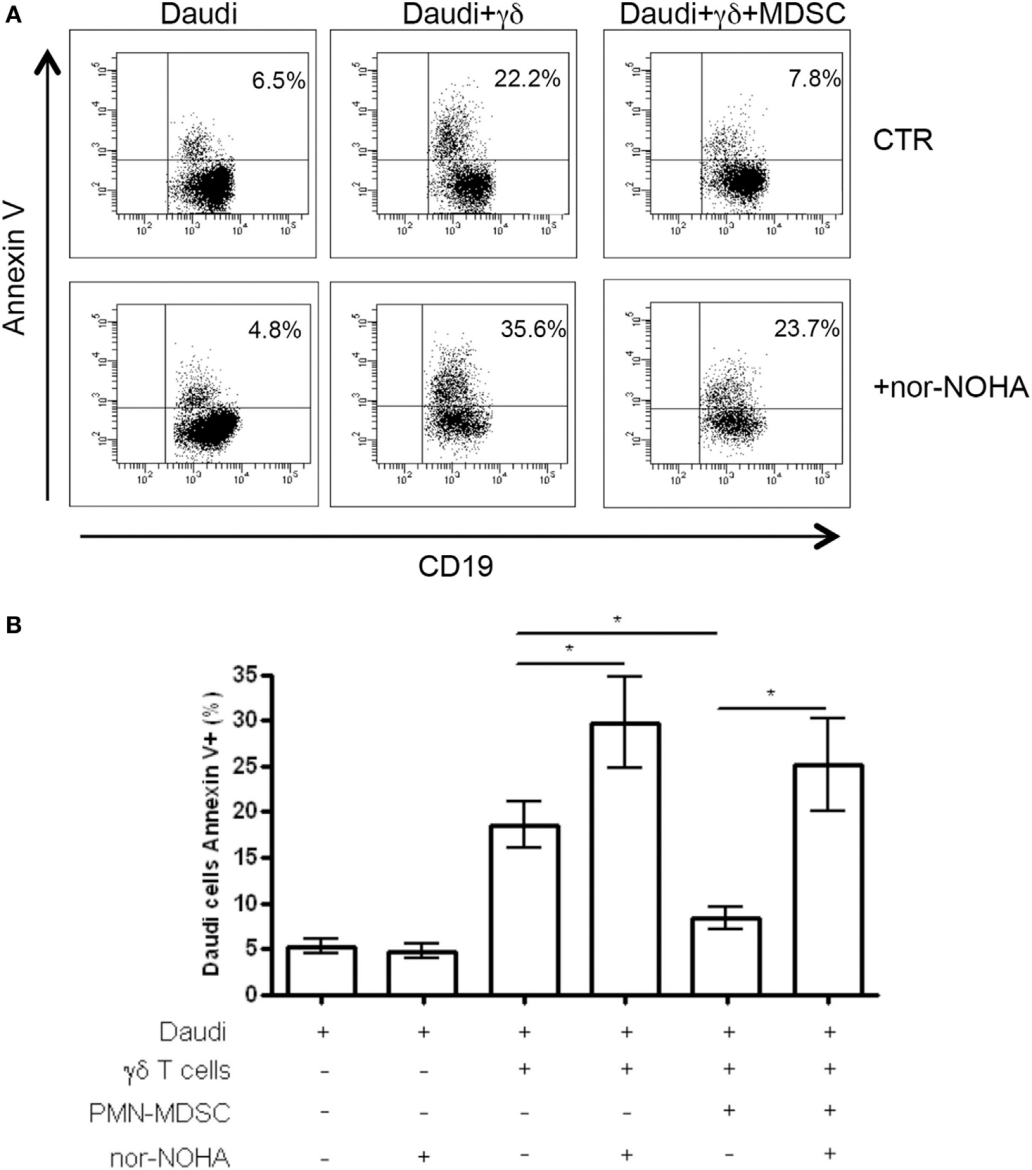
ArgI is involved in Vδ2 T suppression. Daudi cells were cultured with purified γδ T cells in the presence of PMN-MDSC (1:2:2 ratio) and 1 mM of nor-NOHA. After 18 h apoptotic (Annexin V+) Daudi cells were evaluated by flowcytometry. **(A)** Representative dot plots showing Annexin V + Daudi cells (CD19+) in the indicated conditions. **(B)** Percentage of Annexin V + CD19 + Daudi cells in the indicated conditions. Results are show as mean + SEM of three independent experiments (*p < 0.05).

To understand the mechanisms used by PMN-MDSC to inhibit γδ T cells cytotoxic activity, we cultured Daudi cells with purified γδ T cells and PMN-MDSC (1:2:2 ratio) in the presence of specific inhibitors of Arginase I (nor-NOHA), indoleamine 2,3-dioxygenase (1-MTD) or nitric oxide sinthases (l-NMMA). We found that 1-MTD and l-NMMA did not affect the capacity of PMN-MDSC to decrease cytotoxic function of γδ T cells (data not shown), suggesting that indoleamine 2,3-dioxygenase and nitric oxide sinthases are not involved. On the contrary, nor-NOHA abrogated the inhibition of cytotoxic function of γδ T cells performed by PMN-MDSC (Figures 6A,B). However, an enhancement of of γδ T cells cytotoxicity was also observed in the absence of PMN-MDSC (Figures 6A,B), suggesting that Daudi cells may produce ArgI. However, since the addition of PMN-MDSC to nor-NOHA treated γδ T cells cultured with Daudi cells did not decrease the capacity of γδ T cells to kill their targets may suggests that PMN-MDSC used ArgI as well.

## Discussion

Vδ2 T cells participate in the early stages of the immune response, in fact they are able to recognize antigens displayed following infection or other forms of stress, and to respond to stress without requiring extensive clonal expansion. Moreover, they are able to shape the subsequent adaptive immunity, representing a bridge between innate and adaptive immune response. Furthermore, Vδ2 T cells crosstalk with different cell types of the innate and adaptive immunity potentiating their activity. Vδ2 T cells are able to give help to B cells in producing antibodies (21), and to induce maturation of DC (22) thus influencing αβ T cell priming. Activated Vδ2 T cells shortly produce huge amount of IFN-γ, TNF-α (5) that play a central role in controlling tumor and infections. A pivotal feature of Vδ2 T cells is the cytotoxic potential against tumor cells (9, 10) and infected cells (23, 24), that participate to control the diseases. However, tumors and microbial pathogens are able to evade the immune system by means different mechanisms. It has been clearly demonstrated that MDSC play a detrimental role during tumor pathologies, and their expansion has been shown during several infections (13, 25, 26). MDSC are able to suppress different cell types [reviewed in Ref. (27)]; however, data on Vδ2 T cells are still lacking. We found that, similarly to αβ T cells (19), PMN-MDSC are able to inhibit IFNγ production by phosphoantigen activated Vδ2 T cells. Differently from αβ T cells, the proliferation of Vδ2 T cells was not affected. It has been suggested that IL-2 may inhibit MDSC functions (28), then we cannot exclude that the use of IL-2 necessary to induce *in vitro* Vδ2 T cell proliferation could alter MDSC suppressive capability. PMN-MDSC induced a decrease of CD107a expression on Vδ2 T cells, indeed the cytotoxic activity against Daudi and Jurkat cells was reduced. The impaired cytotoxicity was independent of the modulation of the major inhibitory and activating NK receptors, suggesting a possible TCR-mediated mechanism.

Myeloid-derived suppressor cell are able to produce different immunosuppressive mediators such as ArgI, iNOS, and IDO, that are all able to induce T cell anergy through different pathways (29). Our data suggest that ArgI may be involved in the inhibition of Vδ2 T cells by PMN-MDSC. Interestingly, ArgI inhibition increased γδ T cells cytotoxity also in the absence of PMN-MDSC, indicating that Daudi cells are able to produce ArgI.

The immunomodulating strategies represent one of the main exciting approaches in the battle against cancer and Vδ2 T cells have been tested as a good target for their wide and potent antitumoral activity. Nevertheless, the suppressive tumor environment could affect these strategies. We showed for the first time that PMN-MDSC inhibit the antitumor activity of Vδ2 T cells, opening a key issue in the context of Vδ2-targeted immunoteraphy. Other studies are mandatory in order to better clarify the molecular mechanisms of these suppression and to propose combined strategies aimed to boost Vδ2T cells circumventing tumor- and MDSC-induced Vδ2 T cells inhibition.

## Author Contributions

ASacchi and CA designed the study and wrote the paper. NT and ASabatini performed the experiments. ASacchi analyzed the data. EC, VB, RC, and GG contributed to analyze data and revise the paper.

## Conflict of Interest Statement

The authors declare that the research was conducted in the absence of any commercial or financial relationships that could be construed as a potential conflict of interest.
